# Mouse Prostate Epithelial Luminal Cells Lineage Originate in the Basal Layer Where the Primitive Stem/Early Progenitor Cells Reside: Implications for Identifying Prostate Cancer Stem Cells

**DOI:** 10.1155/2013/913179

**Published:** 2013-06-01

**Authors:** Jianjun Zhou, Lionel Feigenbaum, Carole Yee, Hongbin Song, Clayton Yates

**Affiliations:** ^1^Department of Biology and Center for Cancer Research, Tuskegee University, Tuskegee, AL 36088, USA; ^2^Institute of Disease Control and Prevention, Chinese Academy of Military Medical Sciences, Beijing 100071, China; ^3^Science Applications International Corporation-Frederick, National Cancer Institute, Frederick Cancer Research and Development Center, Frederick, MD 21702, USA; ^4^Dermatology Branch, National Cancer Institute, NIH, Bethesda, MD 20892, USA

## Abstract

Prostate stem cells are thought to be responsible for generation of all prostate epithelial cells and for tissue maintenance. The lineage relationship between basal and luminal cells in the prostate is not well clarified. We developed a mouse model to trace cell fate and a mouse model with a slowly cycling cell label to provide insight into this question. The results obtained indicate that putative mouse prostate stem cells are likely to reside in the basal layer.

## 1. **Introduction**

The prolonged use of androgen deprivation therapy is associated with a decline in responsiveness to treatment of prostate cancer treatment, an effect that generally results in a poor clinical outcome. Thus, a focus on the origin of cells capable of developing the androgen-independent tumor mass subsequent to hormonal therapy is of interest.

 Normal prostatic epithelium is comprised of a stratified structure with secretory luminal cells that are androgen-responsive and an interspersed minor population of highly proliferative, androgen-receptor- (AR-) negative basal cells. These AR-negative cells are thought to give rise to AR-positive luminal cells [1]. As determined with human prostate stem-like cells purified from the basal compartment, basal cells can generate luminal cells *in vitro* [2]. Other markers, such as the keratin expression pattern, have also yielded information regarding a subpopulation of stem cells/progenitor cells. Luminal and basal populations are identifiable through expression of specific keratins. Basal cells express keratins 5 and 14 only, but, if luminal cells develop, they lose these keratins and express keratins 8 and 18 [3–5]. 

 The traditional model of prostate epithelial differentiation proposes that adult prostate stem cells are contained within the basal layer and give rise to progenitor/stem cells and androgen-independent, transit-amplifying cells, which can differentiate into luminal cells. This concept is supported by the isolation of intermediate cells, which express both luminal and basal makers, keratin 5 and keratin 18, and by the differentiation of these cells down the luminal lineage [6]. In animal models of castration-induced regenerating prostate, basal cells preferentially survive androgen ablation; however, a subset of luminal cells demonstrate castration-resistance [7]. As determined with a prostate-specific antigen (PSA) CreERT2-based genetic lineage marking/tracing model, a subset of luminal cells not only survive, but they also retain capability for regeneration after castration [8]. While these studies highlight the regenerative capacity of preexisting luminal cells, the origin of regenerative luminal cells is still unclear, since both luminal and basal cell populations survive after castration. 

As presently reported, two approaches were used to determine the lineage-derived fate of basal cells: (1) tracking that allows lineage-specific tagging of the keratin 14-expressing population of cells throughout prostate development in adult mice, and (2) a keratin 5 H2BGFP-label retaining model demonstrating that slowly cycling cells are represented in only a subfraction of keratin 5-expressing cells after hormone/castration manipulation. These cells have the capacity to give rise to more differentiated luminal cells. Results with these models, which suggest that the putative adult stem cells, which have a slowly-cycling feature, most likely reside in the basal layer and are positive for keratin 5 and keratin 14, clarify some of the basic aspects of the biology of the rodent prostate gland.

## 2. Materials and Methods

### 2.1. Cell Fate Tracing Assay

Keratin 14-CreER^tam^ transgenic mice (STOCK Tg (KRT14-cre/Esr1)20Efu/J), LacZ reporter mice (B6.129S4-Gt (ROSA)26Sortm1Sor/J), and EGFP reporter mice (B6; 129-Gt (ROSA)26Sortm2Sho/J) were purchased from the Jackson Laboratory, Bar Harbor, ME, USA. The two transgenic mouse lines were crossed, and double transgenic markers of Cre and LacZ were confirmed by PCR with genomic DNA isolated from their progenies. Tamoxifen (Sigma-Aldrich) was prepared at 100 mg/mL in ethanol, heated to 60°C to dissolve the powder, and then diluted 10 times with sunflower oil aided by 2 min vortex plus sonication for an additional 30 min. Tamoxifen was used to induce Cre activity in gaining its access to the nuclear compartment from the cytoplasm. The administration to young (3 weeks old or younger), double-transgenic mice, of 0.5 mg tamoxifen was accomplished by intraperitoneal injections daily for 5 days. The double-transgenic mice were sacrificed after two cycles of prostate involution (2 weeks) and regeneration by castration and by supplementation (for 2-3 weeks) with testosterone pellets (12.5 mg sustained release, Innovative Research of America). The prostates were removed, and frozen sections from dorsal and ventral glands were obtained. The presence of β-galactosidase was demonstrated with X-gal by use of a commercial kit (Invitrogen).

### 2.2. The Label-Retaining Assay

Transgenic mice expressing a Tet-OFF tTA regulatory transactivator under the control of a keratin 5 promoter were generously provided by Adam Glick (National Cancer Institute; The Pennsylvania State University). These mice were bred to TRETight-H2B/GFP transgenic mice that we created in the NCI-Frederick animal facility. Expression of H2B/GFP is naturally turned on in the double-transgenic mice (keratin 5-tTA:TRETight-H2B/GFP) following development and can be turned off in the basal epithelium of the prostate following the application of doxycycline. A wash-out period is required to identify the label-retaining cells. The prostate epithelial cells that are still H2B/GFP+ after the wash-out period mark the label-retaining cells, which, because of their slowly cycling feature, should represent prostate epithelial stem cells. To ensure the observation of those rare putative stem cells resistant to label dilution due to cell turnover, this wash-out period continued throughout the multiple cycling (over 10) of the prostate epithelium between regression and expansion by hormonal manipulation. After the label-retaining assay of the murine prostate was complete, the animals were anesthetized, and the prostates were removed for histologic analysis.

### 2.3. Castration and Hormone Manipulation

The hormonal manipulation consisted of alternating 2-3 week periods of androgen deprivation and restoration. This manipulation results in regression of the prostate epithelium to a residual basal layer (androgen deprivation) followed by expansion to a multilayered epithelium (androgen restoration). Hormonal deprivation was accomplished by castration, and androgen restoration was achieved by use of implantable testosterone pellets (12.5 mg sustained release, Innovative Research of America), which maintained a serum level of 4 ng/mL. 

### 2.4. β-Galactosidase Staining

The β-galactosidase staining kit was used according to the manufactures suggested procedure to demonstrate the activity of LacZ. Briefly, thawed cryosections of mouse prostates were placed in the fixative solution for 10 min. After washing, the samples were incubated at 37°C with a solution containing X-gal, and the blue cells were assessed within 2 h. The slides were counterstained with nuclear fast red solution. 

### 2.5. Immunofluorescence

For marker expression, standard indirect immunofluorescence staining was performed on 6 *μ*m mouse tissue cryosections. Briefly, the thawed sections were fixed with 4% paraformaldehyde in PBS for 10 minutes at room temperature and, after washing with PBS, blocked with 10% donkey whole serum in PBS. After washing, cells were incubated sequentially with primary antibody rabbit polyclonal anti-mouse keratin 5 (Covance Research Products, Inc) and Alexa Fluor-594 or 546-conjugated donkey-anti-rabbit secondary antibody (Invitrogen), 4′,6-diamidino-2-phenylindole (Sigma) with washing after each step, and mounting medium (Vector). Rabbit anti-mouse keratin 18 (clone LE61) was a gift from E.B. Lane (University of Dundee, Dundee, UK). Standard direct immunofluorescence was performed similar to the above procedures with EGFP detection, except that the only antibody used was Alexa Fluor 594 conjugated rabbit anti-GFP (Invitrogen). The images were acquired and analyzed with a Zeiss Axio fluorescence imaging system (Carl Zeiss MicroImaging Inc.).

## 3. Results

### 3.1. Tracing of Basal Cell Development in Adult Mouse Prostate via Keratin 14

**Figure 1:**
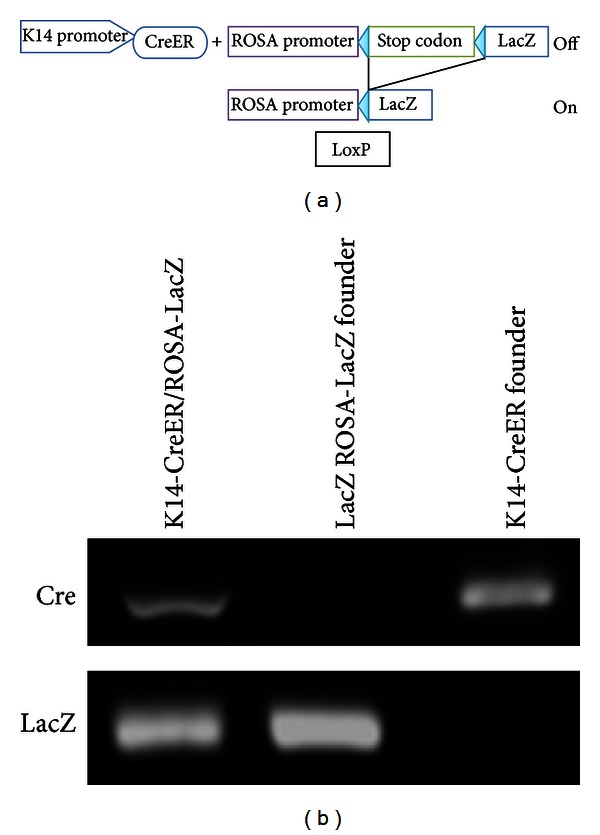
Mouse prostate basal lineage development in mice, as determined by the cell fate tracking system. (a) Schematic representation of the generation of keratin 14-CreER/ROSA-LacZ mouse model. (b) PCR for CRE and LaZ expression in the keratin 14-CreER/Rosa-LacZ confirmed the successful generation of transgenic mice.

 Analysis of keratin expression during prostate development has been useful in distinguishing undifferentiated basal cells and, during tissue regeneration, the more differentiated luminal cells. To determine if luminal cells, which constitute the major population (90%) in mouse prostate epithelia, are derived from basal cells during injury repair, the lineage tracing Cre/loxP system was utilized to track cells expressing keratin 14 (a marker of basal cells) and their differentiated progenies in the mouse prostate. These transgenic mice contain the tamoxifen-inducible Cre gene (CreER) under the control of the keratin 14 promoter. To visualize the developmental and regenerative pattern of keratin 14 expressing-cells, keratin 14-CreER mice were crossed with ROSA 26-LacZ mice. ROSA 26 is a universal promoter, in this case driving a LacZ gene preceded by a floxed stop sequence [9] (Figure 1(a)). For both CRE and LacZ expression, crossing was verified by PCR (Figure 1(b)). Primers for both CRE and LacZ are listed in Table 1. Crossing ROSA 26-LacZ mice with keratin 14-CreER mice and subsequent dosing with tamoxifen led to recombination of the Rosa 26 minigene, with loss of the stop sequence and expression of the LacZ gene.

**Figure 2:**
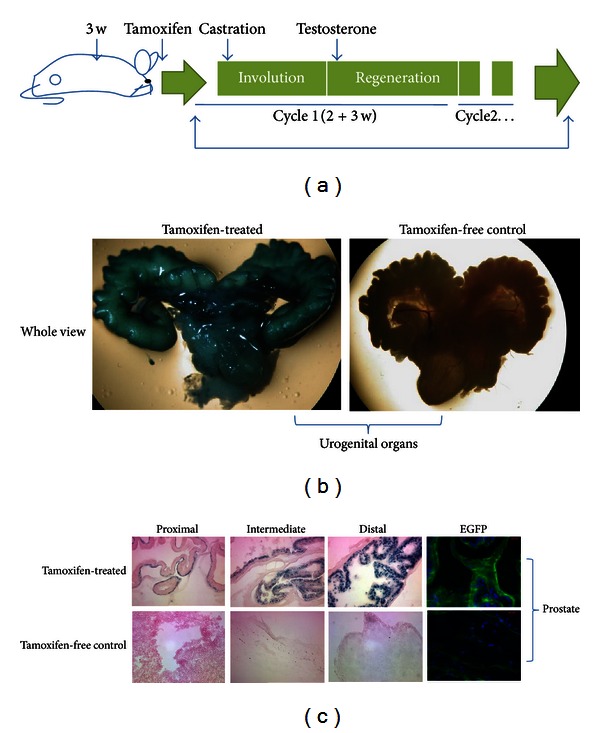
Lineage tracking of keratin 14 expression in developing prostate mouse nodules. (a) Schematic representation of the treatment protocol. (b) β-Galactosidase staining, or fluorescent imaging of the genetic label controlled by basal cell-specific keratin 14 gene promoter activity, showed up in all cell lineages of mouse prostate nodules before and after treatment of the mice with tamoxifen. (c) The more differentiated distal prostate region showed a higher density of β-galactosidase staining than that in the proximal and intermediate regions. The magnification of the microscopy was 400x.

 Before being sacrificed, tamoxifen-treated keratin 14-CreER/ROSA-LacZ mice underwent 2 cycles of androgen manipulation by castration and testosterone supplementation (Figure 2(a)). Experimental and control mice were stained with X-gal to visualize LacZ gene expression. The keratin 14-CreER genetic-based labeling efficiency was evident in tamoxifen-treated mice, as no X-gal staining was observed in untreated mice (Figure 2(b)). Tamoxifen-induced, keratin 14 expressing-basal prostate cells gave rise to a full lineage of cells in the adult prostate nodule. LacZ expression was evident in the luminal cells in both the distal and intermediate regions, with essentially no staining in the basal cell layer, indicating that keratin 14-positive basal cells gave rise to luminal cells (Figure 2(c)). In contrast, LacZ expression was observed in the proximal region, and expression was contained with the basal layer of cells. Untreated keratin 14-CreER/ROSA-LacZ mice did not exhibit any positive staining. Thus, it appears that, in the intermediate and distal regions, keratin 14-expressing cells adopt a luminal fate and are exclusively retained in the basal layer in the proximal region. 

### 3.2. Slowly Cycling Cells within the Proximal Region Are Keratin 5^+^/Keratin 18^−^

**Figure 3:**
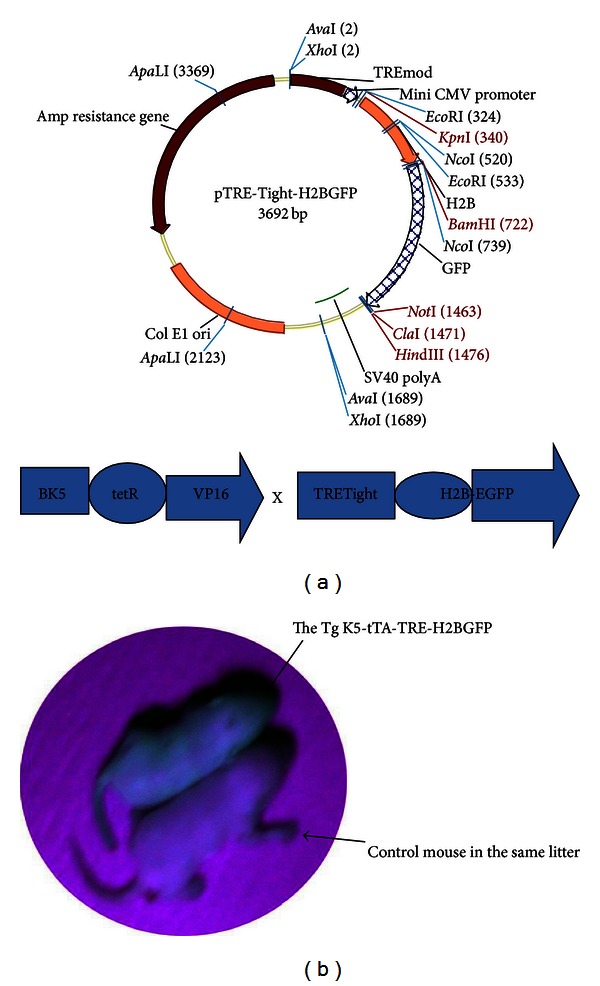
Generation of double transgenic mice keratin 5-tTA: TREtight-H2B-EGFP. (a) The transgenic construct was generated by inserting an H2B-GFP fused gene fragment into the pTRE-Tight vector. (b) Schematic representation of generation of the double-transgenic mice by crossing keratin 5-tTA and TREtight-H2B-EGFP mice. (c) Epifluorescence of a whole animal under UV light. Only the double-transgenic mice with keratin 5-tTA: TREtight-H2B-EGFP showed green skin under UV light.

 The accepted paradigm for prostate development is that all epithelial cells are derived from a common embryonic precursor that expresses keratin 5 [10]. Experiments involving use of bromodeoxyuridine- (BrdU-) based label-retaining assays demonstrate that mouse prostatic epithelial stem cells are concentrated in the proximal region of prostatic ducts, where keratin 5-expressing basal cells are highly enriched [11]. However, a major limitation in the use of BrdU to assess label retention is that the presumed slow turnover of stem cells may prevent these cells from incorporating the label. In addition, it is not possible to test the function of cells prospectively isolated based on their BrdU content. To circumvent these problems, we generated a keratin 5-tTA-TRE-H2BGFP mouse strain that allows for tightly controlled, ubiquitous, doxycycline-inducible expression of an H2B-GFP fusion protein with the keratin 5 promoter upstream (Figure 3(a)). Since skin keratinocytes are also derived from basal keratin 5-expressing cells, the “green skin” can be detected by UV light, further demonstrating a double transgenic individual mouse with H2BGFP-labeled mouse prostate epithelium (Figure 3(b)).

**Figure 4:**
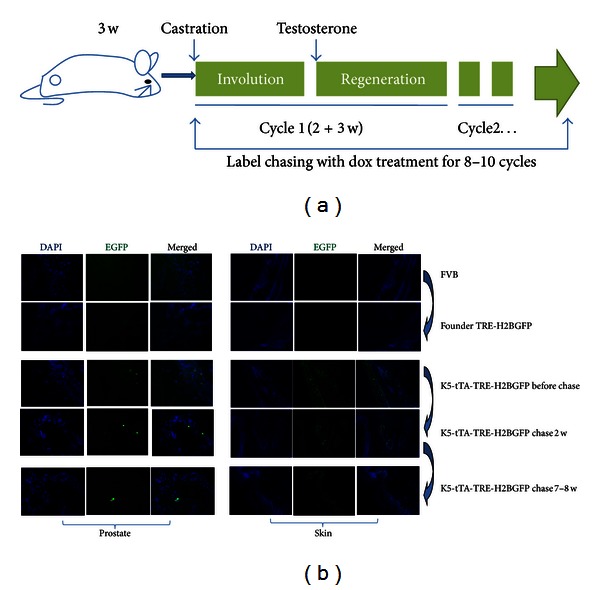
Keratin 5-expressing cells in the proximal region of prostate tissue after multiple cycles of androgen deprivation and restoration. (a) Schematic representation of the labeling protocol in Keratin 5-tTA-TRE-H2BGFP mice. (b) Mice were sacrificed at the end of the chase and full cycles of label wash-out (involution and regeneration of more than 10 cycles) and tissue sections from the proximal regions of prostate were imaged for endogenous GFP expression indicating the K5 positive cells. Skin tissue was utilized as a positive control.

**Figure 5:**
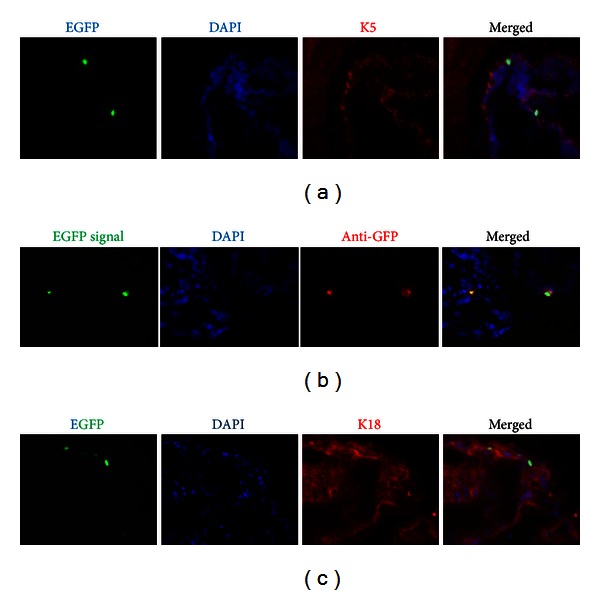
In the basal layer of transgenic prostates, H2BGFP-labeled, slowly cycling cells are keratin 5-specific, not keratin 18-specific. (a) Fluorescent microscopy confirmation of H2BGFP tet-off labeling and chase in the prostate of the double transgenic mice. Mouse tissues containing H2BGFP-labeled, slowly cycling cells, costained with keratin 5 antibody, were specific to the basal layer. (b) The H2BGFP label in mouse tissue was confirmed by a GFP-specific antibody. (c) In the transgenic prostate, H2BGFP-labeled, slowly cycling cells were not keratin 18-specific, as determined by costaining with keratin 18 antibody.

 Keratin 5 expression was tracked through multiple cycles of androgen manipulation, by castration of young mice and by injection of testosterone pellets into adult mice (Figure 4(a)). Keratin 5-tTA-TRE-H2BGFP mice were sacrificed at the end of the chase and full cycles of label wash-out (involution and regeneration of more than 10 cycles). Tissue sections of the proximal region were imaged, with sections of skin as negative controls (Figure 4(b)). Cells that retained the GFP label were rare; however, all positive cells were contained within the basal layer (Figure 4(b)). The GFP signal was specific, for immunofluorescence with an antibody to GFP (anti-GFP) demonstrated virtually an identical staining pattern (Figure 4(b)). To assess the keratin expression pattern further, application of antibodies to keratin 5 and keratin 18, markers of basal and luminal cells, respectively, was also conducted. As determined with differential color immunofluorescence, most cells within the basal layer are keratin 5 positive (red); however, colocalized, immune-stained keratin 5/endogenous keratin 5-GFP cells are still rare (Figure 5(a)). This was confirmed by anti-GFP and Alexa 594 antibodies as positive controls (Figure 5(b)). These cells were also negative for expression of keratin 18 (Figure 5(c)), which further suggested that these slowly cycling cells had features of stem cells. Results with the keratin 5-tet-off-H2BGFP model, which were consistent with the conclusion from data derived with the model for genetic tracing, keratin 14-CreER/ROSA-LacZ, reinforce the concept that, although rare, the renewable cells that survive androgen manipulation are found in the proximal region and are derived from the basal layer. 

## 4. Discussion

 Stem cell biology and tumor biology are intimately related. An understanding of the function of prostate stem cells in the lineage-associated differentiation responsible for maintaining tissue integrity has potential application to prostate regeneration and tumorigenesis. Previous attempts to define these lineage-derived cells have utilized assays performed *in vitro* and *in vivo*; murine models have been used predominately. In contrast to human prostate, which has a complete prostate tissue unit with a continuous basal and luminal layer, the mouse prostate has four major lobe pairs and is further divided into numerous ducts. There is no clear basal layer in the ducts. Results from previous investigations utilizing the label-retaining assay suggest that the proximal region of the mouse prostate is more likely to contain adult prostate stem cells that express basal markers and that the distal region of the duct contains more differentiated luminal cells, with expression of luminal makers [11, 12]. In contrast, there is evidence to support a luminal origin of cells for normal prostate regeneration as well as prostate cancer stem cells [13, 14]. Nevertheless, there remains a lack of agreement as to which cells are responsible for prostate development.

 To identify stem/progenitor cells capable of generating multiple lineages, we utilized a multidirected approach. First, we were interested in identifying cells within the basal layer and determining which basal-derived cells were capable of luminal differentiation. Keratin 14 is a specific marker of basal cells. As established with the mouse model of tamoxifen-induced, label-retaining keratin 14-CreER/ROSA-LacZ, the initial keratin 14-mediated LacZ expression was eventually restored in the luminal cells, as opposed to basal cells in the distal and intermediate regions. However, within the proximal region, keratin 14 expression was primarily expressed in the basal layer. Based on these findings, we propose that, during prostate development, especially in the scenario of tissue injury, luminal cells are derived from the basal layer. However, since most, if not all, of the basal cells retained keratin 14 expression, these cells are likely to be the progenitor cells, not the stem cells, within the basal layer that give rise to keratin 14-expressing luminal cells. Thus, our results agree with other reports that the basal layer is the origin of more differentiated luminal cells. 

 Multiple lines of evidence support the hypothesis that stem cells within the basal layer of prostates in noncastrated and castrated animals are responsible for tubule-forming capacity and give rise to multipotent progenitor cells within the basal layer. By use of an artificial tissue injury and repair process, the presence of living keratin 5-positive stem cells was tracked in keratin 5-tTA-TRE-H2BGFP mice, with the hypothesis that the luminal lineage is derived from a basal lineage. Furthermore, the slowly cycling cells that retain the H2BGFP label after multiple cycles of castration/hormone replacement would represent the population of adult prostate stem cells. The keratin 5 GFP-positive cells were only a minor fraction of the cells. These GFP-positive, keratin 5-expressing cells also lacked coexpression of luminal marker, keratin 18. This suggests that all other cells, including transit-amplifying cells and possibly some progenitor/stem cells entering into the cell cycle, which lose H2BGFP expression, are diluted out. Since keratin 5 is a marker of basal cells, the results indicate that these cells are basal cells, or at least that they are derived from the basal layer. 

 With keratin 5 as a marker, others have shown that the proximal cells of mouse prostatic ducts are slow cycling, have a high proliferative potential, and give rise to large, branched glandular structures that produce prostatic secretory products [11]. Nevertheless, we cannot be sure that keratin 5^+^/keratin 18^−^ cells are prostate stem cells, since it is impossible to determine distant lineage with this approach. Our evidence does suggest that these cells are basal-restricted, slowly cycling progenitor cells. Further evidence is required to characterize this population of cells. 

 We present strong evidence that the basal-derived lineage is responsible for prostate development and tissue regeneration, but there are indications that luminal cells retain regenerative capacity after animals are castrated. The most recent is a report of experiments involving use of a PSA-CreERT2-based genetic lineage tracing system. These results demonstrate that, in a mouse model, preexisting luminal epithelial cells survive and proliferate during cycles of regression and regrowth of adult prostate [15]. Although keratin 5-positive cells were evident by immunostaining, none co-localized with PSA-expressing cells that were responsible for AR-positive luminal cells. While the different number of regenerative cycles could be responsible for these divergent results, it is not clear if the observed luminal cell regeneration is the result of basal-derived luminal progenitor cells that become active only during an immediate response to injury/repair. Furthermore, there may be, within the basal and luminal compartments, stem/progenitor cells with the capacity to regenerate prostate tubules after the animals are castrated. Our findings that keratin 14-expressing cells with restricted label are expressed in luminal cells during mouse development points to a contribution of stem cell/progenitor cells within the basal compartment during development and regeneration. Further evidence, derived with double-knockout, conditional-expression models with basal and luminal differentiation targets, is required to determine which population of cells is responsible for tissue regeneration. 

 The present findings have implications in initiation of prostate cancer. Basal cells from primary benign human prostate tissue, with the cooperative effects of AKT, ERG, and AR, recapitulate the histological and molecular features of human prostate cancer, with loss of basal cells and expansion of luminal cells expressing PSA and alpha-methylacyl-CoA racemase *in immunodeficient mice* [16, 17]. In PTEN-null mice, there is a preferential expansion of basal cells relative to luminal cells [18], and these cells demonstrate more efficient capacity for cancer initiation relative to luminal cells. A role for basal cells in prostate cancer has been implicated during treatment as well. Most prostate cancers are treated with hormone ablation therapy, with eventual relapse of hormone-insensitive cells. Thus, hormonal therapy may interfere only with the bulk population of AR-expressing cells, while the AR-negative, more primitive tumor initiating cells survive hormonal therapy and give rise to progeny that have developed a mechanism to escape hormonal therapy. Thus, further characterization of the population associated with tumor initiation could lead to more effective therapies. 

 In summary, with the use a mouse model for tracking cell fates and a mouse label-retaining assay, we determined that, in the prostate, luminal cells are derived from a basal lineage and that slowly cycling cells, which may represent adult prostate stem cells, reside in the basal cell compartment. These observations have implications for the concept of epithelial cell lineage in the developing human prostate and for identification of cancer stem/initiating cells. 

## Figures and Tables

**Table 1:** Primers for Genotyping for K14-CreER/Rosa-LacZ mice.

Primers	Sequence 5′ → 3′
LacZ mutant forward	GCG AAG AGT TTG TCC TCA ACC
LacZ wild type Forward	AAA GTC GCT CTG AGT TGT TAT
LacZ wild type Reverse	GGA GCG GGA GAA ATG GAT ATG
Cre forward	GCG GTC TGG CAG TAA AAA CTA TC
Cre reverse	GTG AAA CAG CAT TGC TGT CAC TT
